# Methylated SEPT9 combined with AFP and PIVKA-II is effective for the detection of HCC in high-risk population

**DOI:** 10.1186/s12876-023-02900-6

**Published:** 2023-07-31

**Authors:** Kepu Zheng, Leiyang Dai, Yingpeng Zhao, Laibang Li, Wang Li, Xibing Zhang, Qiuming Su, Ruichao Wu, Yizhou Jiang, Yonglin Chen, Jianghua Ran

**Affiliations:** 1 Department of Hepato-Biliary-Pancreatic Surgery, The Affiliated Calmette Hospital of Kunming Medical University, The First People’s Hospital of Kunming, Kunming, Yunnan 650000 China; 2 Department of Clinical laboratory, Kunming Children’s Hospital, Kunming, Yunnan 650000 China

**Keywords:** SEPT9, Septin9, Hepatocellular carcinoma, HCC, Screening, Hepatic cirrhosis, AFP, PIVKA-II, DCP

## Abstract

**Background:**

The methylation SEPT9 (mSEPT9) appeared to be effective for hepatocellular carcinoma (HCC) detection. However, its performance in high-risk population has not been validated. We designed a pilot study and aimed to investigate the performance of mSEPT9, AFP, PIVKA-II and their combination in hepatic cirrhosis (HC) population.

**Methods:**

A training cohort was established including 103 HCC and 114 HC patients. 10 ml blood was collected from each patient with K_2_EDTA tubes, and 3–4 ml plasma was extracted for subsequent tests. The performance of mSEPT9, AFP, PIVKA-II and their combination was optimized by the training cohort. Test performance was prospectively validated with a validation cohort, including 51 HCC and 121 HC patients.

**Results:**

At the optimal thresholds in the training cohort, the sensitivity, specificity and area under curve (AUC) was 72.82%, 89.47%, 0.84, and 48.57%, 89.92%, 0.79, and 63.64%, 95.95%, 0.79 for mSEPT9, AFP and PIVKA-II, respectively. The combined test significantly increased the sensitivity to 84.47% (P < 0.05) at the specificity of 86.84% with an AUC of 0.91. Stage-dependent performance was observed with all single markers and their combination in plasma marker levels, positive detection rate (PDR) and AUC. Moderate correlation was found between mSEPT9 and AFP plasma levels (r = 0.527, P < 0.0001). Good complementarity was found between any two of the three markers, providing optimal sensitivity in HCC detection when used in combination. Subsequent validation achieved a sensitivity, specificity and AUC of 65.31%, 92.86%, 0.80, and 44.24%, 89.26%, 0.75, and 62.22%, 95.27%, 0.78 for mSEPT9, AFP and PIVKA-II, respectively. The combined test yielded a significantly increased sensitivity of 84.00% (P < 0.05) at 85.57% specificity, with an AUC at 0.89.

**Conclusions:**

The performance was optimal by the combination of mSEPT9, AFP, PIVKA-II compared with any single marker, and the combination may be effective for HCC opportunistic screening in HC population.

**Supplementary Information:**

The online version contains supplementary material available at 10.1186/s12876-023-02900-6.

## Introduction

Hepatocellular carcinoma (HCC) is the fifth leading cause of morbidity and the third leading cause of mortality in the world [1]. Although the incidence and mortality of HCC have decreased in the past decades [2], the 5-year survival rate of liver cancer was only 12.1% (2015) [3]. The most effective way to cure HCC is to detect the lesions when they are still curable, and give radical treatment [4, 5]. Therefore, effective screening and early intervention are very important for the prevention and treatment of HCC. Among current screening methods for HCC, ultrasound combined with AFP is still the standard screening method recommended by guidelines and consensus [6, 7]. However, ultrasound (US) is easily affected by the operator’s skills, experience, and the degree of obesity of subjects. It was reported that the sensitivity of screening using US alone or combined US/AFP reached 47% and 63%, respectively, for early-stage HCC (BCLC 0-A) [8]. It was also reported that the compliance of US screening was only 46.87% for a single screening, and was as low as 7.3% for six consecutive screening [9, 10]. This compromised the screening capability of US or combined US/AFP screening. Therefore, accurate, fast, convenient and cost-effective screening method is still needed to facilitate HCC screening.

There are many known risk factors for HCC, including HBV infection, HCV infection, nonalcoholic fatty liver disease (NAFLD), alcohol abuse, smoking, and aflatoxin intake, etc. The efficiency of HCC screening in these populations is not high, partially due to the low cancer incidence and low compliance. Therefore, screening targeting populations with higher HCC risk could be more efficient with high compliance. It was reported that 80–90% of HCC patients undergo hepatitis→hepatic cirrhosis→precancerous disease→HCC route for HCC development, especially for those with hepatitis B/C virus infection [11–14]. An annual HCC incidence of 3–6% was reported for those with confirmed diagnosis of hepatic cirrhosis, and an annual HCC incidence of > 6% was reported for those with confirmed diagnosis of hepatic cirrhosis with nodules [11–16]. Patients with hepatic cirrhosis (HC) represent a subgroup of population with much higher risk for HCC than those without cirrhosis. It was estimated that HBsAg positive HC patients had 18.5 times higher risk than HBsAg positive patients without HC [11]. Since HC patients generally receive anti-virus therapy or liver protecting treatment in clinics or hospitals, opportunistic screening of HC patients at hospital environment may be an efficient way identifying those with high HCC risk.

In recent years, liquid biopsies based on cell-free DNA (cfDNA) have emerged as a promising noninvasive cancer screening method for clinical applications [17]. cfDNA can detect a variety of potential cancer markers, such as mutation [18], methylation [19, 20], copy number change [21], etc. Several studies have demonstrated the feasibility of liquid biopsy to detect mutations and methylation in HCC screening by next-generation sequencing (NGS) [21–25]. Many more studies demonstrated the applicability of single markers or their combination in HCC screening by quantitative polymerase chain reaction (qPCR) and immunoassays [26–28]. The most representative studies investigated the combination of methylated SEPT9 (mSEPT9) and AFP, which showed significantly higher sensitivity than any single marker alone [26–28]. However, these studies were mostly case-control, and did not reflect the performance of combined markers in screening scenario.

In this study, we aimed to combine one methylation marker (mSEPT9) with two protein markers (AFP and PIVKA-II) to enhance the detection capability in HC population. These markers are well-known in HCC detection with high sensitivity and/or specificity from previous studies [27–34], but screening study has not been performed with mSEPT9 or its combination with AFP and PIVKA-II in HCC high-risk population. By establishing training and validation cohorts of HCC high-risk population, we hoped to optimize the performance of the combination in this pilot study and provide evidence for future large-scale screening study.

## Methods and materials

### Ethics

The study plan and ethics materials were submitted to the ethics committee of the affiliated Calmette hospital of Kunming medical university (the first people’s hospital of Kunming) before recruitment of patients and tests started. The study was approved by the hospital ethics committee. Informed consent was obtained from all patients before the collection of blood samples and all patients were informed the test results.

### Study design, patients, and confirmation of diagnosis

**Fig. 1:**
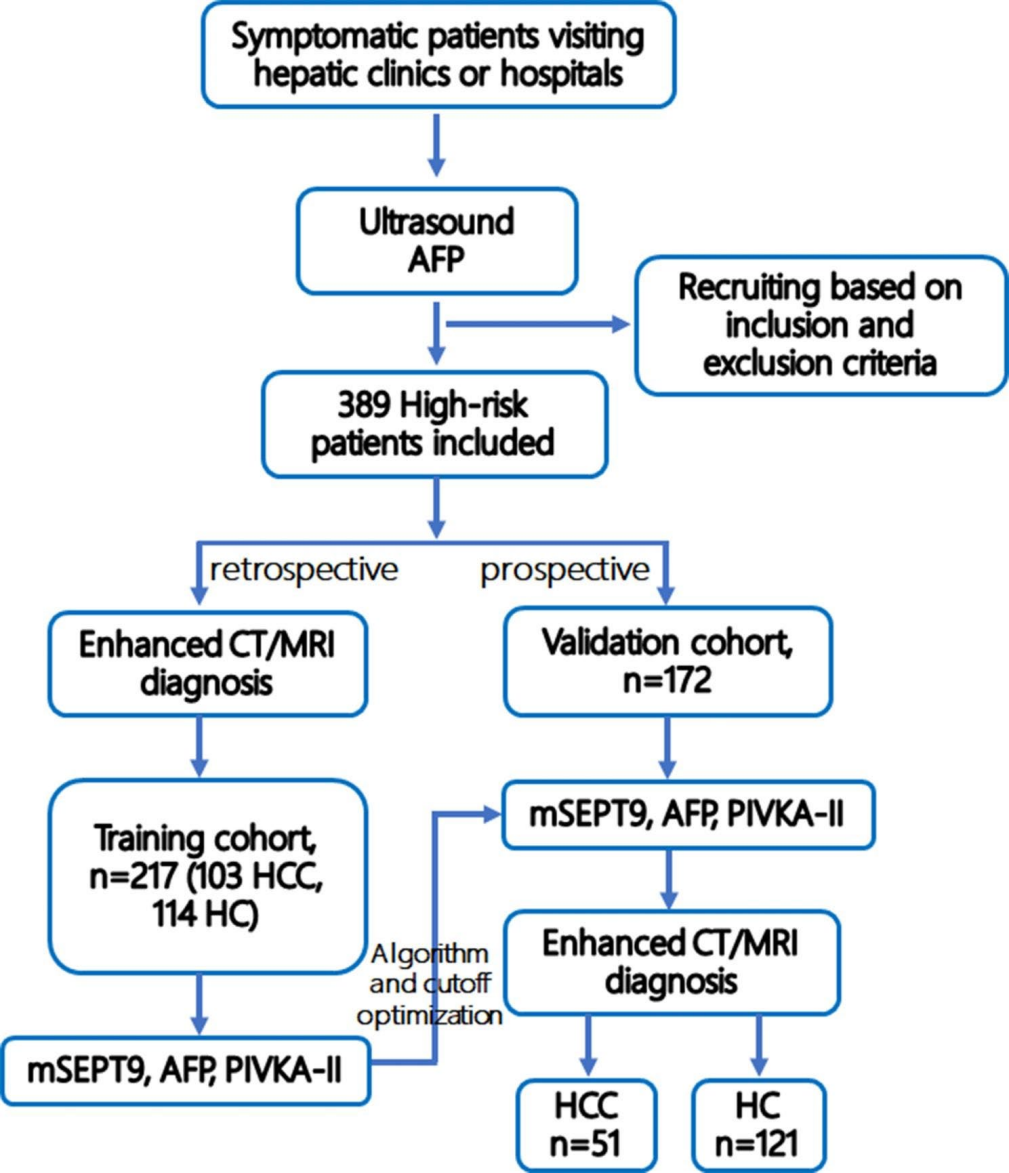
The flowchart for patient recruitment, tests and diagnosis for both training and validation cohorts in this study. Symptomatic patients visiting haptic clinics or hospitals all received ultrasound and AFP test as initial screening, and 389 high-risk patients were included based on inclusion and exclusion criteria. Patients in the training cohort was retrospectively recruited based on the diagnosis from enhanced CT/MRI examination, and was tested by mSEPT9, AFP and PIVKA-II(DCP) before any treatment for this visiting. Patients in the validation cohort was prospectively recruited and were tested by mSEPT9, AFP and PIVKA-II(DCP) before CT/MRI diagnosis, and they were divided into HCC and HC groups based on the diagnosis

The study was designed and performed in the affiliated Calmette hospital of Kunming medical university (the first people’s hospital of Kunming). For test training and validation, both training cohort and validation cohort were established (Fig. 1). The recruitment of patients for training cohort applied a case-control design. The clinical status of HCC and HC patients was determined by imaging and/or pathological examinations before blood draw for all tests, and blood samples were obtained from all outpatients and inpatients who met the selection criteria before any treatment (Fig. 1). Several departments were involved in patient recruitment, including the infection department, the hepatobiliary surgery department and the oncology department. The main inclusion criteria include: adults over 18 years old with complete clinicopathological information and confirmed diagnosis of HCC or HC by enhanced computed tomography (CT) or magnetic resonance imaging (MRI) and/or subsequent pathological examination. The main exclusion criteria include: pregnant woman, history of any cancer, or history of liver transplant or blood transfusion in the past three months and those with incomplete clinical information.

The recruitment of patients for validation cohort was prospective before the clinical diagnosis of HCC and HC was determined. The main inclusion criteria include: adults over 18 years old with defined HCC high risk or very high risk, including those with HC, HC with diabetes or first-degree relative HCC family history, those with HC nodules or low-grade or high-grade dysplasia nodule (LGDN or HGDN), etc. [15, 16]. The main exclusion criteria include pregnant woman, history of any cancer, or history of liver transplant or blood transfusion in the past three months and those with incomplete clinical information: Patients with Child-Pugh B or C were also excluded from the study and only Child-Pugh A patients were recruited, as Child-Pugh B or C involving hepatic functional deficiency may affect the marker performance. Practically, all patients who visited the hospital were examined by ultrasound. patients were enrolled with reference to ultrasound results, in which those with suspected HC, HC with nodules, hepatic precancerous disease and HCC were involved (Fig. 1). Apart from ultrasound, HC patients were also selected based on the results of liver stiffness measurement (Fibroscan). Those with suspected HC or HCC were recruited for blood draw. Blood samples were obtained from all outpatients and inpatients who met the selection criteria before any treatment. The diagnosis was not confirmed before the blood draw. Several departments were involved in patient recruitment, including the ultrasound department, infection department, the hepatobiliary surgery department and the oncology department. The diagnosis of all patients was determined after blood draw by enhanced CT or MRI and/or subsequent pathological examination (Fig. 1).

As a result, a total of 217 subjects, including 103 HCC and 114 HC, were recruited for the training cohort, and a total of 172 patients, including 51 HCC and 121 HC, were recruited for the validation cohort (Table 1; Fig. 1). For prospective cohort study, all technicians who transferred the blood samples or performed the tests were blinded to the information of potential diagnostic status of patients. Investigators were also blinded to the ultrasound and CT/MRI results during the study.

**Table 1:** A summary of demographic and clinicopathological information for patients involved in this study

Factors	Categories	Training cohort			Validation cohort			P value
		HCC	HC		HCC	HC		
Sex								
	male	78	80		36	94		0.534
	female	25	34		15	27		
Age								
	< 40	5	5		3	8		0.974
	40–49	18	19		9	23		
	50–59	32	38		17	41		
	60–69	34	37		19	39		
	≥ 70	14	15		3	10		
Disease history								
	HBV	83	90		43	99		0.773
	HCV	7	8		5	11		
	Alcoholic	5	5		2	6		
	others	8	11		1	5		
Family history of HCC								
	Yes	28	22		15	28		0.419
	No	75	92		36	93		
HCC stage								
	BCLC 0-A	38			25			0.334
	BCLC B	45			17			
	BCLC C-D	20			9			
Liver function status								
ALT (U/L) (mean(SD))		42.3 (18.2)	33.8 (15.0)		45.6 (23.1)	36.3 (12.3)		0.14
AST (U/L) (mean(SD))		44.0 (22.3)	51.6 (23.0)		43.2 (25.6)	48.2 (18.9)		0.44
albumin (g/L) (mean(SD))		35.9 (19.1)	34.4 (18.9)		37.5 (20.2)	36.8 (16.5)		0.36
total bilirubin (µmol/L) (mean(SD))		19.4 (13.6)	17.9 (11.5)		18.7 (14.8)	19.0 (10.1)		0.95
platelet counts (×10^9^/L) (mean(SD))		158.8 (74.0)	115.6 (54.3)		152.6 (81.0)	120.8 (48.5)		0.94
prothrombin time (s) (mean(SD))		12.9 (3.3)	15.7 (4.5)		13.3 (4.3)	14.9 (4.4)		0.68
Total		103	114		51	121		

HCC: hepatocellular carcinoma; HC: hepatic cirrhosis; HBV: hepatitis B virus; HCV: hepatitis C virus; BCLC:Barcelona clinic liver cancer; ALT: alanine aminotransferase; AST: aspartate aminotransferase; SD: standard deviation

### Sample size estimation

Sample size estimation was based on the equation N = Z^2^*[p (1-p)]/E^2^, for known detection sensitivity, in which Z is a statistical parameter (Z = 1.96 for 95% CI), and E represents the error (10% was chosen in this study), and p represents the putative positive detection rate. The p value in this study for HCC was obtained from previous studies reporting the sensitivity of the mSEPT9, AFP and PIVKA-II tests in HCC. If the p value was set to 0.85, an estimated 49 HCC cases were required. The HC subjects were recruited in at least 1:2 to that of the HCC cases (Table 1).

### Sample collection and storage

Samples were collected from outpatients and inpatients of the designated departments, and the sample information was recorded. A 10 ml peripheral blood sample was collected with a 10 ml K_2_EDTA anticoagulant tube (Jiangsu KANGJIAN Medical Apparatus Co., Ltd, Taizhou city, Jiangsu province, China). Plasma was isolated from the blood samples by spinning the tube at 1350 g for 12 min. The supernatant was collected in a 15 ml centrifugal tube and spined at 12,000 g for 12 min. The final supernatant was collected in a 15 ml centrifugal tube for cfDNA extraction. 200 μl plasma was aliquoted for AFP and PIVKA-II tests. All plasma samples were transported to an authenticated clinical laboratory and stored at -80 °C for future tests.

### DNA extraction and qualitative PCR analysis of SEPT9, and test for AFP and PIVKA-II

Plasma cfDNA extraction and bisulfite conversion were performed following the manufacturer’s instructions of the mSEPT9 assay (BioChain (Beijing) Science and Technology, Inc., Beijing, China). One PCR was performed for each subject on an ABI 7500 Fast Dx Real Time PCR device (Life Technologies, Thermo Fisher Technology (China) Co., LTD, Shanghai, China). ACTB was used as an internal reference to assess the integrity of each sample. The validity of mSEPT9 test results for each sample was determined on the basis threshold count (Ct) values of ACTB, the positive and negative controls. Plasma AFP and PIVKA-II levels were measured using the corresponding commercial kits with the Abbott ARCHITECT i2000SR chemiluminescence immunoanalyzer, according to manufacturer’s instructions (Abbott Laboratories; Chicago, IL, USA).

### Data analysis, interpretation, and determination of threshold

Data interpretation and analysis of mSEPT9 data followed the instructions for use from the manufacturer, as previously described [27, 35]. A relative methylation value was determined for each sample using the ΔΔCt method as previously described [27, 36]. Analyses including the χ2 test, student t-test, linear correlation analysis and receiver operating characteristic (ROC) curves were performed and figures were plotted with the Graphpad PRISM 5.0 software (GraphPad Software, Inc, La Jolla, CA 92,037, USA). The comparison of the area under the curve (AUC) was performed by the DeLong’s test using the MedCalc program (www.medcalc.org).

The thresholds for mSEPT9, AFP and PIVKA-II were determined by identifying the optimal Youden’s index (sensitivity + specificity-1) from the ROC analysis. The blood levels of markers at the best Youden’s index were determined as the thresholds. The establishment of thresholds also referred to the scatter plots. The determination of the final thresholds took into account both Youden’s index and scatter plots. The thresholds for mSEPT9 and the combined test were adjusted in the validation cohort using the identical methods. The combined test of the three markers was determined as positive if any one of the three markers was positive. A score for combined test was calculated based on the average percentile of mSEPT9, AFP and PIVKA-II for a subject. For example, the percentile for one patient was 45% for mSEPT9, 38% for AFP and 78% for PIVKA-II, the average percentile is (45 + 38 + 78)/3 = 53.67.

## Results

### Combination of mSEPT9, AFP and PIVKA-II enhanced the detection performance of HCC

**Fig. 2:**
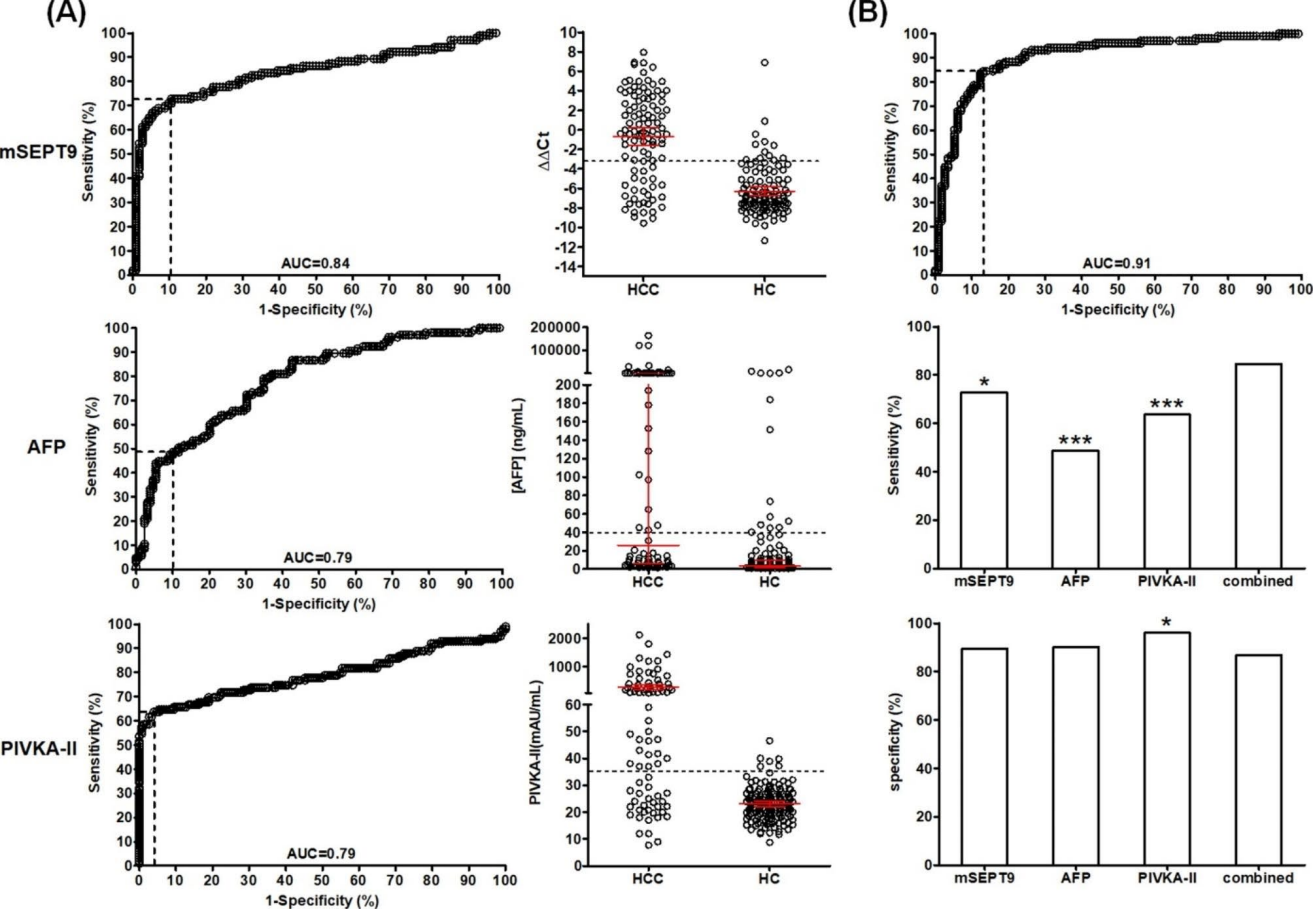
The performance of mSEPT9, AFP and PIVKA-II and their combination in HCC detection in the training cohort. **Panel A**: the ROC curves and scatter plots for mSEPT9(upper lane) and AFP(middle lane) and PIVKA-II(lower lane). The dashed lines indicate the optimal sensitive and specificity based on the calculation of Youden’s index. AUC is labeled on each panel. The dashed lines in scatter plots indicate the optimized threshold for positive/negative interpretation. The bars in the scatter plots represent mean with 95% confidence interval. **Panel B**: The ROC curve for combined test and comparisons of detection sensitivity and specificity. The dashed lines ROC curves indicate the optimal sensitive and specificity based on the calculation of Youden’s index. The values of sensitivity and specificity in panel B were from a series of single calculations based on interpretation of sample test results of the corresponding groups. Results of statistics of sensitivity and specificity calculated from ROC curves are shown in Table 2. The asterisks indicate the significance from Chi-square test when compared with the combined group. *:P < 0.05; ***:P < 0.001

In order to systematically examine the performance of mSEPT9, AFP and PIVKA-II in HCC detection in HCC high-risk population, we first established a training cohort by recruiting 103 HCC and 114 HC patients following the inclusion, exclusion criteria and the recruiting flow chart (Table 1; Fig. 1). The thresholds of mSEPT9, AFP and PIVKA-II were determined by identifying the best balancing point between the positive and negative detection, as shown in Fig. 2A and described in the method section (Youden’s index). At the optimal thresholds shown in Table 2 for each marker, the sensitivity, specificity and AUC was 72.82%, 89.47%, 0.84, and 48.57%, 89.92%, 0.79, and 63.64%, 95.95%, 0.79 for mSEPT9, AFP and PIVKA-II, respectively (Table 2; Fig. 2A). No significant difference was found among AUCs of the three markers. The combined test of the three markers was determined as positive if any one of the three markers was positive. Using the simple algorithm, the combined test achieved a sensitivity of 84.47% at the specificity of 86.84% with an AUC of 0.91 under the combined score threshold at 35.0 (Table 2; Fig. 2B). The combined AUC was significantly higher than that of the mSEPT9 (P = 0.033), AFP (P = 0.0012) and PIVKA-II (P = 0.0057), although no significant difference in AUC was found among mSEPT9, AFP and PIVKA-II. In addition, the combination of mSEPT9 and AFP also showed a significantly higher AUC (0.89) than mSEPT9 (P = 0.046) or AFP (P = 0.001). The sensitivity and specificity for the three markers and combined test were compared in Fig. 2B. The sensitivity of the combined test was significantly higher than mSEPT9 (χ^2^ = 4.162, P = 0.041), AFP (χ^2^ = 29.833, P < 0.0001) or PIVKA-II (χ^2^ = 12.147, P = 0.00049). The combined specificity had no significant difference to that of mSEPT9 and AFP, although significant difference was found with PIVKA-II (χ^2^ = 5.481, P = 0.019).

**Table 2:** Performance of the mSEPT9, AFP, PIVKA-II and combined tests in training cohort

	mSEPT9	AFP	PIVKA-II	combined
Sensitivity (%, 95%CI)	72.82 (63.16–81.12)	48.57(38.70-58.53)	63.64 (53.36–73.07)	84.47 (76.00-90.85)
Specificity (%, 95%CI)	89.47 (82.33–94.44)	89.92(83.38–94.52)	95.95 (91.39–98.50)	86.84 (79.23–92.44)
AUC (95%CI)	0.84 (0.78–0.90)	0.79 (0.73–0.84)	0.79 (0.72–0.86)	0.91 (0.86–0.95)
Threshold	ΔΔCt=-3.0	40.0 ng/ml	35.0 mAU/ml	combined score = 35.0

AFP: alpha fetal protein; PIVKA-II: protein induced by vitamin K absence or antagonist II; CI: confidence interval; AUC: area under curve; Ct: cycle threshold

**Fig. 3:**
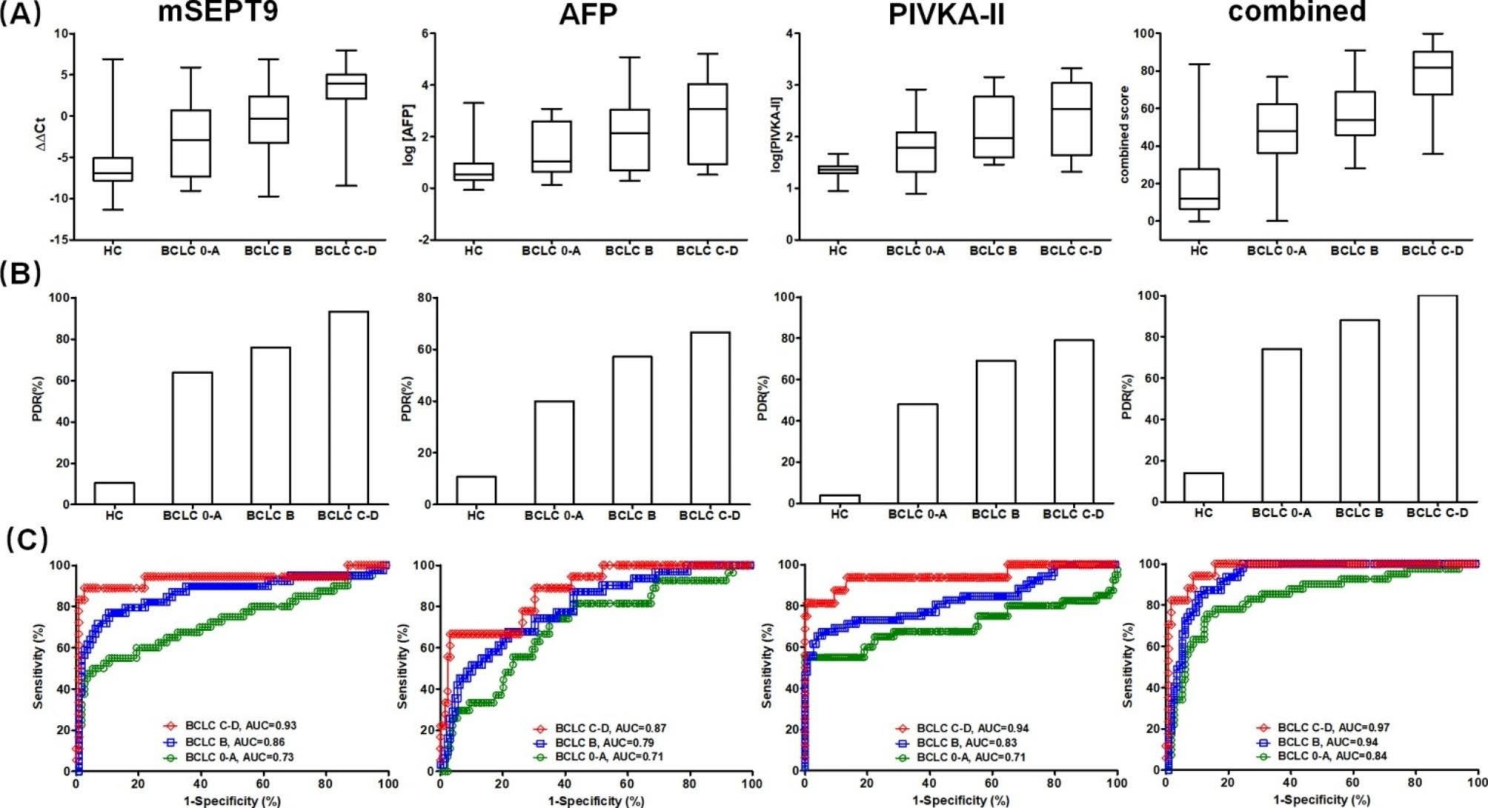
Stage-dependent performance of mSEPT9, AFP and PIVKA-II and their combinations in HCC detection in the training cohort. Data for HC is shown for comparison. **Panel A**: box and whisker plots for quantitative data of each stage in mSEPT9, AFP, PIVKA-II and their combination. **Panel B**: the positive detection rate (PDR) for each stage for all tests and their combination. **Panel C**: the ROC curve for each stage for all test and their combinations

We further studied the stage-dependent performance of the markers and their combination. Figure 3 shows the quantitative measurements (Fig. 3A), the positive detection rate (Fig. 3B) and ROC curves (Fig. 3C) for BCLC stage 0-A, B and C-D. The data for HC was also shown for comparison. It can be observed that the ΔΔCt values of mSEPT9, the AFP and PIVKA-II plasma levels and the combined score all exhibited stage-dependent trend, in which higher stages showed higher levels of markers (Fig. 3A). Correspondingly, the PDR of all markers and combined test also exhibited dose-dependent trend, and higher stages appeared to have higher PDR (Fig. 3B). This trend was also reflected in the ROC curve. In all markers and combined test, higher stages exhibited bigger AUC than lower stages (Fig. 3C).

**Fig. 4:**
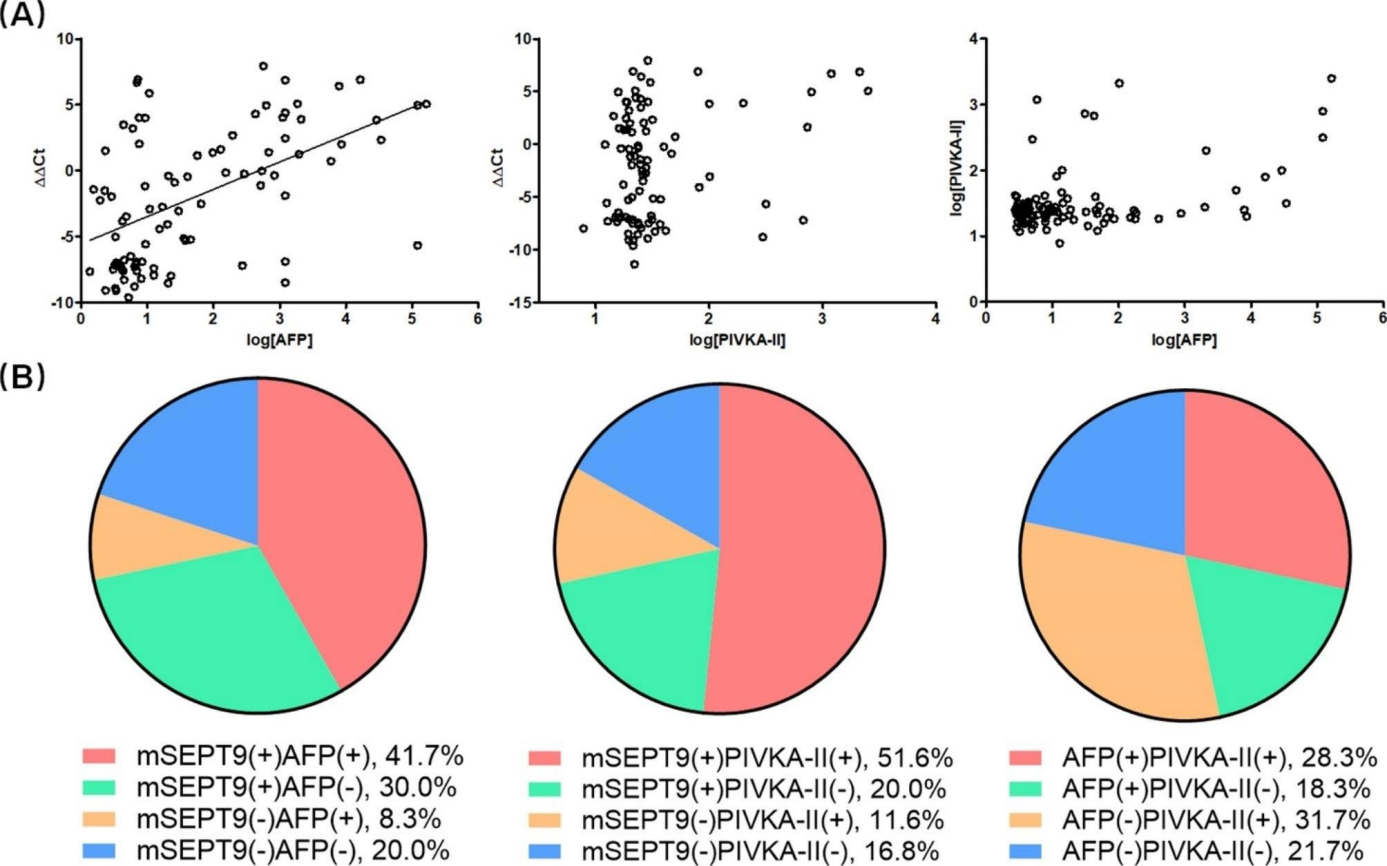
The correlation and complementarity of mSEPT9, AFP and PIVKA-II. **Panel A**: moderate correlation was found between mSEPT9 and AFP (r = 0.527, P < 0.0001), while no significant correlation was found in quantitative measurements between mSEPT9 and PIVKA-II, and between AFP and PIVKA-II. **Panel B**: the complementarity between the tests is shown by the percentage of four situations. Strong complementarity can be observed between any two of the three markers

The correlation and complementarity of all three markers was investigated next. The correlation between markers was studied first. It can be seen from Fig. 4A that moderate correlation was found between mSEPT9 (ΔΔCt values) and AFP (log[AFP]) (r = 0.527, P < 0.0001). In contrast, no significant correlation was found in quantitative measurements between mSEPT9 and PIVKA-II, and between AFP and PIVKA-II. The complementarity of markers was studied next, and Fig. 4B shows significant complementarity between single markers. mSEPT9 detected an extra 30% of patients in AFP negative patients, and detected an extra 20% of patients in PIVKA-II negative patients. Interestingly, AFP and PIVKA-II also showed good complementarity, in which AFP detected an extra 18.3% of patients in PIVKA-II negative patients, and PIVKA-II detected an extra 31.7% of patients in AFP negative patients. This observation suggested that any two of the three markers exhibited good complementarity, providing optimal sensitivity in HCC detection when used in combination.

### Combination of mSEPT9, AFP and PIVKA-II is effective in the detection of HCC

**Fig. 5:**
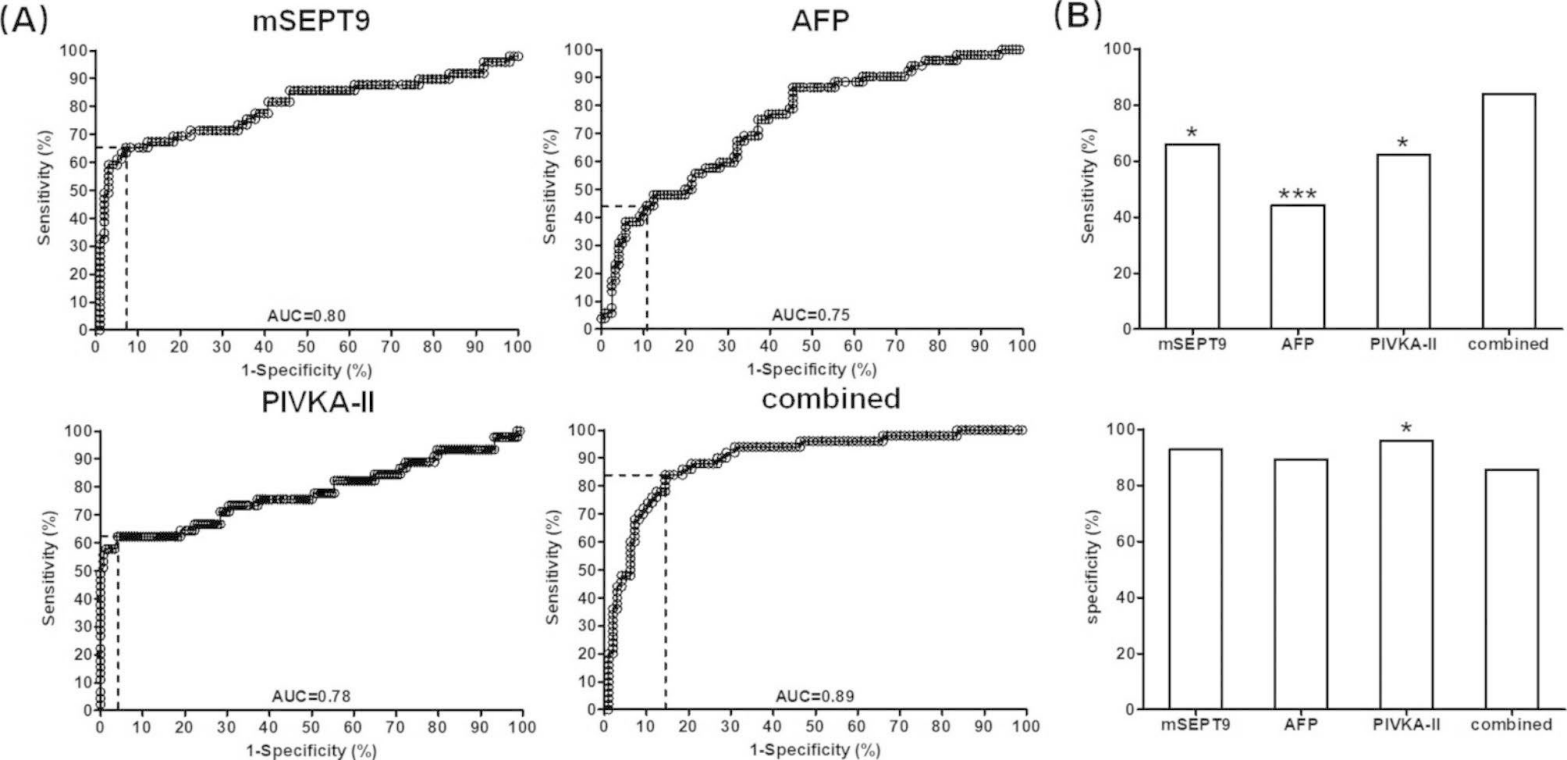
The performance of mSEPT9, AFP, PIVKA-II and their combinations in validation cohort. **Panel A**: the ROC curves for the tests and their combination. The dashed lines indicate the optimal sensitivity and specificity after validation. AUC is labeled on each panel. **Panel B**: the sensitivity and specificity for all tests and their combinations in validation. Statistics of sensitivity and specificity calculated from ROC curves are shown in Table 3. The asterisks indicate the significance from Chi-square test when compared with the combined group. *:P < 0.05; ***:P < 0.001

A validation cohort including 51 HCC and 121 HC patients was established prospectively to validate the performance of the three markers and their combination. As shown in Table 3; Fig. 5A, the threshold of mSEPT9 was adjusted from ΔΔ=-3.0 to ΔΔ=-2.7 based on the test results and the optimal balance between sensitivity and specificity (optimal Youden’s index). The threshold for combined detection was adjusted accordingly from 35.0 to 38.0. The threshold for AFP and PIVKA-II remained unchanged. The validated sensitivity, specificity and AUC was 65.31%, 92.86%, 0.80, and 44.24%, 89.26%, 0.75, and 62.22%, 95.27%, 0.78 for mSEPT9, AFP and PIVKA-II, respectively. No significant difference was found in AUC among the three markers. The combined test achieved a sensitivity of 84.00% at 85.57% specificity, with an AUC of 0.89. The combined AUC was significantly higher than that of the mSEPT9 (P = 0.034), AFP (P = 0.0045) and PIVKA-II (P = 0.032). In addition, the combination of mSEPT9 and AFP also showed a significantly higher AUC (0.88) than mSEPT9 (P = 0.048) or AFP (P < 0.001). The sensitivity and specificity for the three markers and combined test were compared in Fig. 5B. The sensitivity of the combined test was significantly higher than mSEPT9 (χ^2^ = 5.162, P = 0.023), AFP (χ^2^ = 17.172, P = 0.00003) or PIVKA-II (χ^2^ = 6.095, P = 0.014). The combined specificity had no significant difference to that of mSEPT9 and AFP, although significant difference was found with PIVKA-II (χ^2^ = 5.813, P = 0.016). It appeared that the performance of mSEPT9, AFP and PIVKA-II was not affected by the etiology of HCC, as no significant difference in sensitivity was found among HBV, HCV, alcoholic and others groups (Supplementary Table 1). This was also confirmed by a pervious study [30].

**Table 3:** Performance of the mSEPT9, AFP, PIVKA-II and combined tests in validation cohort

	mSEPT9	AFP	PIVKA-II	combined
Sensitivity (%, 95%CI)	65.31 (50.36–78.33)	44.23(30.47–58.67)	62.22 (46.54–76.23)	84.00 (70.89–92.83)
Specificity (%, 95%CI)	92.86 (85.84–97.08)	89.26(82.33–94.15)	95.27 (90.50-98.08)	85.57 (76.97–91.88)
AUC (95%CI)	0.80 (0.71–0.89)	0.75 (0.67–0.83)	0.78 (0.68–0.88)	0.89 (0.84–0.95)
Adjusted threshold	ΔΔCt=-2.7	40.0 ng/ml	35.0 mAU/ml	combined score = 38.0

AFP: alpha fetal protein; PIVKA-II: protein induced by vitamin K absence or antagonist II; CI: confidence interval; AUC: area under curve; Ct: cycle threshold

## Discussion

mSEPT9 has been found as an effective biomarker for the detection of CRC in blood [27, 37]. It has been approved as a CRC screening test by the US FDA and a CRC detection test by regulatory agencies of other countries [37, 38]. The blood mSEPT9 was also found to be positive in other cancers, including hepatocellular carcinoma [27, 30, 31], esophageal cancer [27, 39, 40], gastric cancer [27, 39, 41, 42] and lung cancer [43–45], etc. Its performance appeared to be optimal in CRC and HCC, and the detection sensitivity was above 70% at 90% specificity [27, 30, 31, 46, 47]. Meanwhile, it appeared that the performance of mSEPT9 in HCC detection was not affected by the etiology of HCC, i.e. HBV, HCV infection or alcoholic cirrhosis, etc. This was confirmed by the report from Oussalah and colleagues [30], in which HCC patients with various etiology were involved. Although good performance of mSEPT9 was found in HCC detection, validation of screening performance in large cohorts has not been performed. This is partly due to the fact that the necessity of HCC screening by blood markers is not as strong as that in CRC. Blood screening test in CRC aims at population that cannot be covered by the invasive colonoscopy screening, while imaging screening by ultrasound/AFP and diagnosis by CT or MRI are non-invasive, which undermines the necessity of blood screening. However, blood screening has its advantages over ultrasound and AFP combined screening. First, ultrasound is easily affected by the operator’s skills, experience, and the degree of obesity of the subjects, while blood test can be standardized without the influence from these factors. Secondly, the sensitivity of ultrasound and AFP was not high enough, and more accurate method is needed to perform efficient screening. It was reported by a meta-analysis that the sensitivity of ultrasound to detect early-stage HCC was 47% and the sensitivity of ultrasound combined with AFP for detecting early-stage HCC was 63% [8]. This result indicated that the screening capability was poor for US and US/AFP combination in early-stage HCC. Thirdly, the compliance of ultrasound and AFP was low, which further compromised the screening capability. As described by retrospective studies, the compliance of US screening was only 46.87% for a single screening, and was only 7.3% for six consecutive screening [9, 10]. This suggested that the US screening capability was largely compromised by the low compliance, although it is a non-invasive convenient test. Therefore, HCC screening by blood test has its application scenarios and target population.

In this study, we aimed at HCC high risk population with diagnosed hepatic cirrhosis. It was estimated that the HCC incidence in this population could be between 3% and 6% [11–16]. Opportunistic screening in this enriched population may therefore identify high ratio of HCC patients. Different to screening in average-risk population or low-middle risk population, this high-risk population included patients with hepatic cirrhosis, hepatic cirrhosis with liver nodules and those with precancerous or cancerous diseases. Screening test in this population therefore needs to distinguish between non-cancerous benign diseases and cancerous diseases, which requires both high sensitivity and high specificity. Therefore, test optimization in this specific population is required, and this was the focus of the present study. The optimization aimed to balance the sensitivity with the specificity, and this was because the false positive rate in this high-risk population was higher than that in the normal subjects or low-middle risk population, especially for methylation markers. The pursuit of high specificity comes at the cost of reduced sensitivity. However, the sensitivity can be improved by combined use of multiple markers, i.e. mSEPT9, AFP and PIVKA-II in this study.

The combination of mSEPT9, AFP and PIVKA-II yielded high sensitivity and specificity by adjusting the thresholds of the three markers. The false positive rate of mSEPT9 was high in hepatic cirrhosis and therefore the threshold was established higher than previously reported [27, 36]. This was also true for AFP, which had a high false positive rate in hepatic cirrhosis. In contrast, the specificity of PIVKA-II appeared to be satisfactory for a diagnostic test, and the threshold in this study was even lower than previously reported [48, 49]. By doing this, the combined use of the three markers still ensured high specificity. On the other hand, we found that the combined use improved the overall sensitivity, especially in the validation cohort, which demonstrated the advantages of combined markers from multiple omics. A simple algorithm was therefore used to ensure both high sensitivity and specificity.

AFP and PIVKA-II have been widely used as markers for HCC detection and recognized as the most effective protein markers. The sensitivity of AFP ranged roughly from 40 to 70% at the specificity of over 90% [32–34], and the sensitivity for PIVKA-II ranged roughly from 50 to 80% at the specificity of over 90% [48–51]. Although AFP or PIVKA-II alone was not potent enough for detecting early-stage HCC, the combination of AFP, AFP-L3 and PIVKA-II increased the detection sensitivity and was suggested to be effective in HCC screening [52–54]. In addition, it was found that the performance of AFP and PIVKA-II for HCV-related HCC [55–57] and alcoholic HCC [58–60] was at similar levels to that found in HBV-related HCC. Meanwhile, AFP and PIVKA-II have also been used as markers for recurrence monitoring, therapeutic response monitoring and prognosis prediction [61–63] in HCC therapy. In our study, we found good complementarity between mSEPT9 and AFP or PIVKA-II, and the involvement of mSEPT9 further enhanced the detection sensitivity beyond the combination of AFP and PIVKA-II. In fact, mSEPT9 exhibited the best balance between sensitivity and specificity for HCC detection among all known single markers [27, 30, 31]. The high sensitivity of mSEPT9 in HCC and CRC made it an optimal marker when used alone or in combination with other markers.

Apart from conventional markers such as AFP and PIVKA-II, the development of aMAP score, Glypican-3 (GPC-3) and golgiprotein73 (GP73) was also shown to be significant in HCC screening. The aMAP score involved age, sex, albumin-bilirubin and platelets, and satisfactorily predicted the risk of HCC development among over 17,000 patients with viral and non-viral hepatitis from 11 global prospective studies [64, 65]. It was reported that the cut-off value of 50 was associated with a sensitivity of 85.7–100% and a negative predictive value of 99.3–100%, and the cut-off value of 60 resulted in a specificity of 56.6–95.8% and a positive predictive value of 6.6–15.7%. The scoring system could help to establish a risk score-guided HCC surveillance strategy [64, 65]. GPC-3 is a member of the glypican family that anchors heparin sulfate proteoglycan on the cell surface with glycosylphosphatidylinositol. GPC-3 is low expressed in normal human tissues, but overexpressed in diseased liver, especially HCC [66]. Di Tommaso et al. [67] found that the sensitivity and specificity of GPC-3 in early HCC diagnosis were 68.75% and 90.91%, respectively, with 91.67% PPV and 66.67% NPV. Golgiprotein73 (GP73) is a membrane protein that is commonly expressed in the epithelial cells of many human tissues and can also be detected in the serum of patients with liver disease. The sensitivity and specificity of serum GP73 in HCC diagnosis were 69-95% and 70-93%, respectively, which were significantly higher than AFP [68]. In addition, there was no significant difference in serum GP73 levels between AFP positive HCC patients and AFP negative HCC patients [68]. A recent meta-analysis of GP73, GPC-3, and AFP in HCC early diagnosis found that the AUC of the combined detection of these three biomarkers was 0.95, indicating that the diagnostic accuracy of the combined detection was higher than that of GP73, GPC-3, and AFP alone or combined [69].

In recent years, liquid biopsy techniques targeting cell-free DNA (cfDNA) have emerged as promising noninvasive screening methods for HCC [17]. cfDNA test by NGS can detect a variety of potential cancer markers, such as mutation [18], methylation [19, 20], copy number change [21] and microRNA [70], etc. Several studies have demonstrated the feasibility of liquid biopsy to detect mutations and methylation in HCC [22–25, 71]. Multi-gene-based targeted sequencing or genome-wide epigenetic signature recognition and subsequent modeling are major strategies for detecting HCC. Based on the targeted methylation sequencing prototype method reported in 2009, a method for specific capture of genomic targets for monomolecular bisulfite sequencing was used to quantify DNA methylation at single nucleotide resolution [72]. One study used the above methods and established a diagnostic and predictive model for HCC with a sensitivity of 85.7% and specificity of 94.3% in the training cohort and a sensitivity of 83.3% and specificity of 90.5% in the validation cohort [19], indicating a good prospect of methylation sequencing in early HCC screening. A number of subsequent studies confirmed that multigene methylation marker detection can be used for HCC screening [31, 73–76]. In the field of epigenetics, in addition to methylation detection, data from hydroxymethylation and microRNA (miRNA) detection methods in HCC screening also showed promising application prospects. Researchers examined genome-wide 5-hydroxymethylcytosine (5hmC) in a large cohort including 2554 Chinese subjects, and established a diagnostic model for early-stage HCC. A 32-gene diagnostic model accurately distinguished early-stage HCC (stage 0/A) from non-HCC (training cohort: AUC = 92.3%; Sensitivity = 89.6%; Specificity = 78.9%; validation cohort: AUC = 88.4%; Sensitivity = 82.7%; Specificity = 76.4%) [77]. Similarly, detection methods based on multiple miRNA markers also appeared to be promising for HCC early screening [70, 78, 79].

The combination of single markers, such as mSEPT9, AFP and PIVKA-II in this study, has its advantages over the NGS sequencing method. First, the tests of single markers are easier than the NGS test in terms of techniques, test operation, lab and instrument requirements and staff training. Secondly, the tests of single markers do not need validation by very large cohorts and complex algorithm, making them easier in clinical trials and practice. Thirdly, the costs of the tests of single markers are much lower than the NGS test currently. Fourthly, the sensitivity and specificity obtained from tests of single markers after optimization are comparable with those obtained from NGS tests, making them highly cost effective.

This study had some limitations. First, validation of the test should be performed in larger cohorts, ideally in prospective screening population in future, as the number of patients in current cohorts in this study was still limited. Secondly, optimization of test performance and algorithm in hepatitis C, alcoholic liver disease, and NAFLD related populations should be performed to facilitate the HCC screening of these populations in future. Thirdly, algorithm can still be optimized in future in larger cohorts.

## Electronic supplementary material

Below is the link to the electronic supplementary material.

Supplementary Material 1

## Data Availability

The datasets generated and/or analyzed during the current study are available from the corresponding author upon reasonable request.
