# Supplementary material for: Methylated SEPT9 combined with AFP and PIVKA-II is effective for the detection of HCC in high-risk population
Source: BMC Gastroenterol. 2023 Jul 31;23:260. doi: 10.1186/s12876-023-02900-6 (PMC10388499; doi:10.1186/s12876-023-02900-6)
Supplement: Supplementary file 1 — Supplementary Material 1 [file 12876_2023_2900_MOESM1_ESM.pdf]

Supplementary Table1. sensitivity of the mSEPT9, AFP, PIVKA-II for various etiology

Cohort	Etiology	n	mSEPT9(%)	AFP(%)	PIVKA-II(%)
training	HBV	83	73.5	50.6	63.8
	HCV	7	71.4	42.9	57.1
	alcholic	5	60	40	60
	others	8	75	37.5	62.5
	overall	103	72.82	48.57	63.64
validation	HBV	43	65.1	46.5	62.8
	HCV	5	60	40	60
	alcholic	2	50	50	50
	others	1	100	0	100
	overall	51	65.31	44.23	62.22
Oussalah et al., 2018	Entire population	51	78.4		
(3 positive triplicates)	HCV-related cirrhosis	15	66.7		
	Alcoholic cirrhosis	30	83.3		

AFP: alpha fetal protein; PIVKA-II: protein induced by vitamin K absence or antagonist II; CI: confidence interval; HBV: hepatitis B virus; HCV: hepatitis C virus
